# Clinical Outcomes of Self-Made Polyurethane-Covered Stent Implantation for the Treatment of Coronary Artery Perforations

**DOI:** 10.1155/2021/6661763

**Published:** 2021-05-17

**Authors:** Xiaoyue Song, Qing Qin, Shufu Chang, Rende Xu, Mingqiang Fu, Hao Lu, Lei Ge, Juying Qian, Jianying Ma, Junbo Ge

**Affiliations:** Department of Cardiology, Zhongshan Hospital, Fudan University, Shanghai Institute of Cardiovascular Diseases, Shanghai 200032, China

## Abstract

**Objectives:**

The present study aimed to investigate the short- and long-term clinical outcomes of self-made polyurethane-covered stents (PU-CS) in patients for the management of coronary artery perforation (CAP) during percutaneous coronary intervention (PCI).

**Background:**

Coronary artery perforation is reckoned as a serious complication in PCI and associated with considerable morbidity and mortality. Covered stents have been used for treating the life-threatening CAP during PCI. But in some catheterization laboratories, no commercial CS is immediately available when there is an urgent need for CS to rescue the coronary rupture site.

**Methods:**

We retrospectively identified 24 patients who underwent 31 self-made PU-CS implantations due to CAP in Zhongshan Hospital, Fudan University, from June 2015 to January 2020.

**Results:**

The total procedural success rate of CS to seal the perforation was 79.2%. Nine patients (37.5%) developed cardiac tamponade, of which 8 patients (33.3%) underwent pericardiocentesis and 4 patients (16.7%) underwent cardiac surgeries. Except for 4 cardiac death cases (16.7%), none of myocardial infarction (MI), target lesion revascularization (TLR), and stent thrombosis (ST) was reported during hospital stay. Data from 22 patients (91.7%) were available at 610.4 ± 420.9 days of follow-up. Major adverse cardiac events (MACE) occurred in 6 patients (27.3%), including 5 cases of cardiac death and one TLR case.

**Conclusions:**

Self-made PU-CS demonstrates high rates of successful delivery and sealing of severe CAP during PCI. Although the in-hospital mortality remains high after PU-CS implantation, the long-term follow-up shows favorable clinical outcomes, indicating the feasibility of PU-CS in treating CAP.

## 1. Introduction

The prevalence of coronary artery perforation (CAP) during percutaneous coronary intervention (PCI) is low, ranging from 0.1% to 0.5% [1, 2]. Remarkably, CAP is an uncommon but life-threatening complication, which increases the risk of considerable cardiac adverse events such as cardiac tamponade, emergent coronary artery bypass grafting (CABG) surgery, and death [3]. With the growing number of PCI in high-risk population and complex lesions, the current incidence of CAP is constantly increasing. And a number of patients with a history of cardiovascular risk factors are considered to be not suitable for cardiac surgical therapies. In cases of dreadful coronary perforations, the implantation of covered stents (CS) can be considered as an alternative to emergency surgery. There are several kinds of commercially available CS, making use of different materials compromising polytetrafluoroethylene (PTFE), polyurethane (PU), and pericardium [4]. Besides, some handmade covered stents using autologous vein/artery or coronary balloon are also used in catheterization laboratory when premounted CS are not available [5]. In the current study, a new self-made PU-CS was developed and used as a bail-out strategy for CAP during PCI, as no commercial CS was immediately available in our center. This study aimed to evaluate in-hospital and follow-up outcomes of patients treated by these self-made PU-CS.

## 2. Materials and Methods

### 2.1. Study Population and Design

Between June 2015 and January 2020, we identified 24 consecutive patients who underwent 31 self-made PU-CS implantations for CAP in our center (Zhongshan Hospital, Fudan University, Shanghai, China). Two experienced interventionalists reviewed all original angiograms to confirm the occurrence of CAP and conducted the classification in accordance with the Ellis classification [6]. This study was approved by our institutional review board and was conducted in accordance with the ethical standards laid down in the Declaration of Helsinki. Informed consent was obtained from all participants included in the study.

### 2.2. Study Device

**Figure 1:**
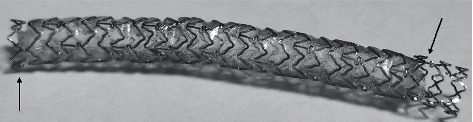
Self-made polyurethane-covered stent. The figure depicts the self-made polyurethane-covered stent covered by 3M™ Tegaderm™ transparent film dressing (arrows).

The self-made PU-CS in the study is shown in Figure 1, and the detailed preparing methods can be found in Supplementary Video 1. Briefly, the self-made PU-CS was made by covering a commercial drug-eluting stent (DES) with PU membrane cutting from 3M™ Tegaderm™ Transparent Film Dressing (Frame Style 1624 W, 3M Company, USA). The length of PU membrane was determined in accordance with DES (at least 2 mm shorter than DES), while the width was usually 8 mm, which ensured the PU membrane was wrapped twice around DES.

### 2.3. Study Definitions and Clinical Outcomes

Coronary lesions were classified following the American Heart Association/American College of Cardiology (AHA/ACC) classification into types A, B, and C [7]. Coronary chronic total occlusion (CTO) was defined as 100% occlusion of a coronary artery with the presence of thrombolysis in myocardial infarction (TIMI) grade 0 flow for more than three months [8]. According to Ellis classification, CAP was defined as follows: type I indicated extraluminal crater without extravasation; type II indicated pericardial or myocardial blush without contrast jet extravasation; and type III indicated contrast jet extravasation through a frank (≥1 mm) perforation, and type III cavity spilling indicated perforation into an anatomic cavity and cardiac sinus [6]. Additionally, Muller and colleagues proposed to add a fifth type of perforation: distal perforation caused by guide wires [9].

The implantation of CS was considered successful after correctly deployed at the perforation sites, achieving residual angiographic stenosis <30%, TIMI grade 3 flow, and no residual extraluminal extravasation and hemodynamic homeostasis [10].

The evaluated in-hospital clinical outcomes consisted of emergent perioperative events (cardiac tamponade, pericardiocentesis, and emergent surgical procedures), cardiovascular and all-cause mortality, myocardial infarction (MI), ischemia-driven target lesion revascularization (TLR), and stent thrombosis (ST). Long-term major adverse cardiac events (MACE) were a composite of cardiovascular and all-cause mortality, MI, and ischemia-driven TLR at follow-up. TLR was defined as ischemia-driven need for any repeat revascularization, either angioplasty or CABG [11]. ST was determined following the Academic Research Consortium definition criteria [12]. We also collected the rate of in-stent restenosis (ISR) via coronary angiography (CAG) or coronary CT angiography (CTA), which was defined as recurrent diameter stenosis >50% within a stent or at its edges [13].

### 2.4. Procedure and Stent Implantation

All patients received a loading dose of aspirin and thienopyridine one day prior to procedure. And unfractionated heparin was administered intravenously to achieve a target activated clotting time of 250–350 s. When CAP was detected by CAG, balloon inflation was usually chosen as the first step to stop extravasation into the pericardium. Cardiac surgery was not given priority when considering its comorbidities and high operative risk. If the contrast streaming still existed, self-made PU-CS was made and delivered to occlude the perforation. When necessary, noncompliant balloon was utilized to do postdilatation within the CS or at their margins. If the extravasation was not halted, additional PU-CS may be implanted to seal the perforation at the operator's discretion. CAG was repeated 20 minutes later to confirm the cessation of extravasation. The immediate postoperative echocardiography was left to the discretion of the operator. All patients received lifetime aspirin of 100 mg/day in the absence of contraindications and thienopyridine therapy as determined by contemporary guidelines.

### 2.5. Data Management

By reviewing our electronic medical records and catheterization lab database, data were retrospectively collected as follows: (1) baseline demographic characteristics; (2) lesion and procedural features; and (3) short- and long-term clinical outcomes data. Long-term follow-up was performed via outpatient clinical visits, inpatient observation, and telephone interviews.

### 2.6. Statistical Analysis

Continuous variables are presented as mean ± standard deviation (SD). Categorical variables are expressed as frequencies with percentages. All analyses were conducted by using IBM SPSS software version 20.0 for Windows (SPSS Inc., Chicago, IL, USA).

## 3. Results

### 3.1. Baseline Clinical Characteristics

Between June 2015 and January 2020, a total of 31 self-made PU-CS were implanted in 24 patients after CAP. The baseline clinical characteristics of these patients are demonstrated in Table 1. The mean age of the population was 68.7 ± 8.4 years, and they were mostly male (81.8%). Almost 40% of the procedures were performed following stable angina whereas approximately 55% cases were for acute coronary syndrome (unstable angina, ST-segment elevation myocardial infarction, and non-ST-segment elevation myocardial infarction).

### 3.2. Lesion and Procedural Characteristics

**Figure 2:**
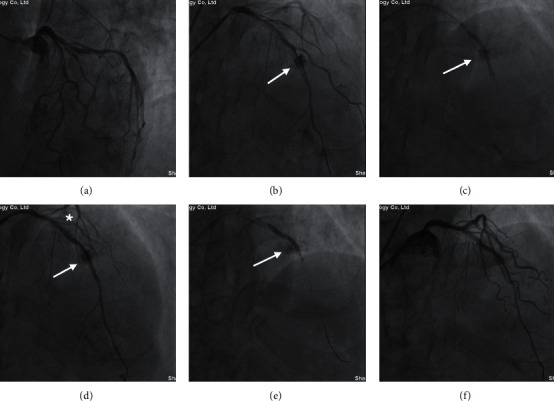
Example of self-made PU-CS implantation to seal a grade III coronary perforation located in LAD. (a) CAG reveals diffuse, tortuous, and calcified lesions in LAD with multiple 80–85% stenotic lesions. (b) CAG demonstrates a coronary artery perforation (Ellis grade III) in LAD (arrow). (c) CAG illustrates the deployment of the first self-made PU-CS (3.0 × 29 mm) in LAD (arrow). (d) CAG shows that the perforation site in LAD persists (arrow) and Guidezilla was used to deliver CS (∗). (e) CAG presents the deployment of the second self-made PU-CS (3.0 × 29 mm) (arrow). (f) Final angiography after successful implantation of CS. PU-CS = polyurethane-covered stent; LAD = left anterior descending coronary artery; CAG = coronary angiography.

**Figure 3:**
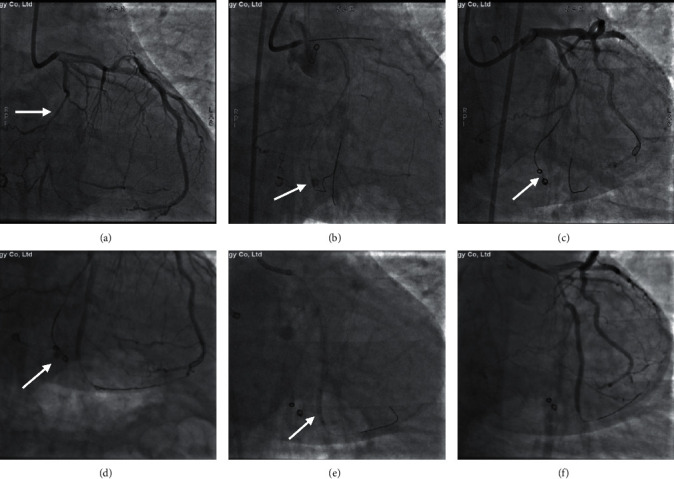
Example of coils and self-made PU-CS implantation to seal a grade II coronary perforation located in the collateral of LCX. (a) CAG reveals LCX-CTO lesion and the collateral vessel of LCX (arrow). (b) CAG shows the coronary perforation in the collateral of LCX caused by guidewire and balloon dilation (arrow). (c) CAG depicts the deployment of two coils (Cook 2.0 × 2.0 mm) (arrow). (d) CAG demonstrates the existing extravasation after coil implantation in the collateral of LCX and stent implantation (Synergy 2.5 × 38 mm & 3.0 × 16 mm) in LCX (arrow). (e) CAG presents the deployment of the self-made PU-CS (2.5 × 18 mm) in the distal segment of LCX to occlude the collateral perforation (arrow). (f) Final angiography after successful implantation of CS. PU-CS = polyurethanecovered stent; LCX = left circumflex coronary artery; CAG = coronary angiography; CTO = chronic total occlusion.

The lesion and procedural characteristics of the study population are presented in Table 2.  Figure 2 provides a typical example of grade III coronary artery perforation in large coronary artery treated by PU-CS.  Figure 3 depicts a case of collateral perforation managed by coils and PU-CS eventually. The majority of the perforated sites treated by CS were localized in the proximal or middle segments of main vessels (83.3%), including 10 in left anterior descending coronary artery (LAD), 5 in left circumflex coronary artery (LCX), and 5 in RCA (right coronary artery). Two perforations (8.3%) were attributed to distal coronary guide wire-induced perforation. The remaining 2 cases (8.3%) occurred in collateral channels during retrograde CTO recanalization.

Patients suffering from type III CAP accounted for 70.8% of all the cases while those of type II CAP constituted 29.2%. Most of the lesions were classified as type B2/C (91.7%) and over half of the lesions were CTO (58.3%). Additionally, the percentage of calcification lesions and lesions at a bifurcation was 41.7% and 33.3%, respectively. Most CAP occurred during balloon postdilatation (33.3%) and stent implantation (20.8%). Intravascular ultrasound (IVUS) was applied in 12.5% patients and Guidezilla™ guide extension catheter (Boston Scientific, USA) was used in 16.7% patients.

The average number of PU-CS used was 1.3 ± 0.5 and the maximum number of PU-CS implanted per patient was 2. The mean stent diameter was 2.7 ± 0.4 mm and the stent length was 21.1 ± 6.8 mm. CS with a diameter below 3.0 mm were implanted in 16 patients, while larger CS (≥3.0 mm) were placed in the other patients. The details of all the artificial PU-CS in the study are provided in Supplementary Table 1.

In this study, 11 coronary perforations were identified during the course of PCI. Although the operators terminated 4 PCI procedures after CS implantation, they managed to proceed the procedure in 7 cases after successful management of CAP by CS implantation and successfully revascularized target vessels. In total, the operators succeeded in ceasing extravasation in 19 cases (79.2%) after the utilization of self-made PU-CS.

### 3.3. In-Hospital Clinical Outcomes

In-hospital clinical outcomes including emergent perioperative events are outlined in Table 3. Eighteen patients (75.0%) experienced pericardial effusion and 9 patients (37.5%) had cardiac tamponade of which 8 patients (33.3%) required pericardiocentesis with relief of tamponade. The remaining patient directly underwent surgical conversion without pericardial drainage.

It was worth noting that the self-made PU-CS failed to occlude the perforations in 5 patients. The soaring pericardial effusion was monitored by the bedside echocardiography in coronary care unit (CCU) in one patient and pericardiocentesis was performed. Then, the patient experienced urgent PCI, which identified CAP. Though receiving self-made PU-CS treatment, the patient died from persistent contrast jet extravasation and cardiogenic shock ultimately. Another patient was found to suffer from cardiac tamponade in the ward after the previous PCI and the condition was gradually restored with successful CABG. The other 3 PU-CS treatment failures underwent an immediate conversion to emergency cardiac surgeries during PCI and 2 of them ended up with cardiac death.

The overall in-hospital mortality rate of the study population was 16.7% (*N* = 4), all associated with cardiovascular death. One patient died due to existing CAP after CS implantation as mentioned above. Another patient died from cardiac rupture related to acute myocardial infarction. The other 2 patients died after emergent cardiac surgery, of which one died due to failed surgical repair during the procedure, and the other died from ventricular fibrillation after successful surgical repair.

### 3.4. Follow-Up Results

Clinical outcomes at a mean follow-up of 610.4 ± 420.9 days are given in Table 4. Two individuals were lost to follow-up and the clinical follow-up was available in 22 patients (91.7%). Follow-up angiography was available in 10 patients (45.5%) of the total with a follow-up of 405.1 ± 226.9 days. Only one death happened after discharge and the total mortality rate increased to 22.7% (*N* = 5) at follow-up. One patient underwent CABG one month after discharge due to recurrent angina after the previous failed PCI. What is more, a total of 2 cases of ISR were observed during angiographic follow-up. One patient with 2.75 × 18 mm PU-CS complained of worsening chest discomfort for 3 months after PCI. ISR was manifested in the distal LAD by CTA, where the PU-CS was implanted. The patient refused to do PCI and HER symptoms were alleviated by conservative medications. Another patient with 2 PU-CS (2.5 × 22 mm and 2.5 × 23 mm) implanted in diagonal branch developed total in-stent occlusion when performing elective CTO recanalization 3 months after CS implantation and was discharged after drug therapy. MI was not identified throughout the entire follow-up period. Supplementary Table 1 provides a detailed description of all the cases in terms of their lesion and procedural characteristics and clinical outcomes.

## 4. Discussion

This is the first investigation focusing on the short- and long-term outcomes of patients receiving self-made PU-CS for the treatment of CAP. The main findings of the current study are summarized as follows. (1) Self-made PU-CS demonstrates high rates of successful delivery and sealing of severe CAP during PCI. (2) Besides large vessel perforation, PU-CS is a feasible option in treating distal wire perforation and collateral perforation in selected cases. (3) Despite the huge burden of in-hospital mortality after PU-CS implantation, the long-term follow-up results were acceptable.

Covered stents have proven to be feasible in various clinical settings including coronary perforations. They can magically create a mechanical barrier to seal the perforation and alleviate the urgent need for emergent surgery [14]. The “sandwich design” PTFE-covered stents (Graftmaster RX Coronary Stent Graft System, Abbott Cardiovascular, USA), which are composed of one PTFE material wrapped between two stainless steel stents, are involved with limited deliverability and feasibility when encountering complex coronary anatomies [15]. Moreover, the performance in the long term was poor due to the high incidence of ISR (25%) and ST (from 3% to 16%) [16–18]. The PK Papyrus CS (Biotronik, USA) was a cobalt-chrome stent platform covered with a single-layer of PU material [19]. Hernandez-Enriquez et al. came to an encouraging conclusion that delivery time of PK Papyrus stent was shorter and brought about reduced frequency of pericardial effusion and cardiac arrest in comparison with the Graftmaster [20]. The largest registry study about PK Papyrus presented there was also a high rate of long-term TLR, mainly due to ISR and ST (9% and 10% at 12 months, respectively) [21]. What is more, second-generation single-layer pericardium-CS (Aneugraft Dx stent, ITGI Medical, Israel) was also a biocompatible and deliverable device to seal the extravasation. Of note, the stent required to be immersed in a wet container of glutaraldehyde solution and then rinsed in sterile physiologic saline before deployment, which was quite inconvenient for the operators in the emergent condition [22]. Nagaraja et al. conducted a systematic review of adverse outcomes for patients receiving Graftmaster, PK Papyrus, and pericardial stents and found that the utilization of PTFE-CS appeared to bring about higher rates of ST (8.9% vs. 4.3% vs. 1.5%, *p*=0.011), pericardiocentesis/tamponade (28.9% vs. 16.0 vs. 20.0, *p*=0.005), and emergency CABG (6.5% vs. 1.2% vs. 1.5%, *p*=0.012) [23]. In spite of fewer ST with pericardium-CS in comparison with others, ISR appeared to occur more frequently in patients with pericardial stents (4.4% vs. 3.0% vs. 21.5%, *p* < 0.001).

Apart from the commercially available CS, the importance of handmade CS in the treatment of CAP cannot be ignored. Autologous vein/artery covered stents possessed prominent advantages in favorable biocompatibility, accelerated endothelialization, and reduced intimal hyperplasia [24]. But the preparation process was involved with surgical operation to obtain autologous vein/artery grafts. As for CS made of an additional new coronary balloon, the fixation methods between balloon and stent and lower deliverability compared to the noncovered stents remained major concerns.

Considering the high likelihood of comorbidities and operative risks, the interventionalists do not prioritize the need for cardiac surgery for the management of CAP. In general, our self-made PU-CS deserves to be considered as a good alternative to cease the extravasation. Firstly, the covering material is a polyurethane membrane widely used in cardiovascular applications, such as heart pump membrane and polymeric valves [25]. Secondly, both DES and transparent film dressing are readily accessible in the Cath Lab and the PU-CS can be manufactured in a short time with the collaboration of operators. Thirdly, our stent provides flexible choices when determining the specific size and number, rather than limited by the promounted stent system. Fourthly, especially in tortuous and calcified lesions, our stent can be deployed to the accurate perforated sites easily and rapidly with or without the aid of Guidezilla for its low crossing profile and high deliverability. Finally, the PCI procedure can continue after the acceptable sealing of the perforation sites, and there is no need to reverse postprocedural standard antiplatelet and anticoagulant strategies.

It was reported that the total procedural success rates for commercial CS usually varied between 90% and 100% [14, 17, 19]. But in Hernandez's study, the procedural success rate of Graftmaster and Papyrus was 69% and 86% (*p*=0.216), respectively [20]. In our study, the new self-made PU-CS achieved sealing of CAP in 79.2% patients, a little bit lower than previously described. The explanation for this may get to the high-risk anatomical features, which were not only related to the higher incidence of CAP but also the severe aftermath of CAP. The percentage of CTO lesions (58.3%) was an issue deserving attention. Furthermore, the high-risk anatomical features were involved in the reduced procedural efficiency of sealing of CAP by the self-made PU-CS.

When compared with similar CS studies with similar sample size, there were striking similarities in the incidence of periprocedural events including pericardial effusion, cardiac tamponade, and pericardiocentesis [10, 18]. But our population presented higher rates of emergent surgery after CAP and in-hospital mortality. This may reflect the combination of population with high burden of cardiovascular risk factors and complex lesions. Reported rates of in-hospital mortality for PTFE-CS in studies involving more patients ranged from 15% to 23% [20, 26] and those of PU-CS were approximately 10% [19, 21]. At 1-year follow-up, figures of all-cause mortality for PTFE-CS ranged from 26% to 41% [16, 20], and those of PU-CS were approximately 26% [20, 21]. According to Nagaraja and colleagues, the long-term mortality for patients receiving Graftmaster, PK Papyrus, and pericardial stents after CAP was 18.5%, 16.0%, and 26.1%, respectively [23]. Pavani et al. conducted an investigation of 102 patients treated with CS (96 with PTFE-CS and 6 with pericardium-CS) after a dreadful grade III CAP and reported that the in-hospital MACE were mainly caused by high in-hospital mortality (14.7%) and acute ST (3.9%) whereas the long-term follow-up was acceptable despite a high ST (6.2%) [26]. And the cardiac tamponade was the critical sign of poor prognosis especially referring to the mortality rate at short-term follow-up [16]. Moreover, a legacy effect of CAP on continued excess mortality between 30 days and 12 months was also reported [27]. In this study, despite the high proportion of complex lesions including CTO, calcification, and bifurcation lesions, the short- and long-term mortality was in line with most previous articles (16.7% and 22.7%, respectively). Reporting only one TLR (4.5%) and no MI during follow-up, the long-term MACE rates for us largely depended on the cardiovascular mortality rates. Hence, it was reasonable to infer that our self-made PU-CS was comparable to other kinds of CS.

In-stent restenosis and stent thrombosis were taken into consideration regarding the use of CS by many interventional cardiologists. Although there is no evidence regarding endothelialization after our PU-CS implantation, excessive neointimal proliferation at the edges of CS has been attributed as a plausible explanation for the restenosis effect according to previous articles [28]. Indeed, previous studies reported that ISR occurred with a frequency of 6%–25% at long-term follow-up [18, 20]. We only observed two ISR cases in 10 patients undergoing angiographic follow-up and both cases were related to CS of small diameter (2.5 mm–2.75 mm). The etiology of thrombotic events might be delayed endothelialization as revealed by intracoronary imaging in the previous study [29]. To our delight, there was an absence of thrombotic event in our investigation after angiographic follow-up. Another reason that may explain the low incidence of ST is that the majority of the perforations were located in large vessels. In the future, large-scale multicenter studies with angiographic and intracoronary imaging follow-up are required to evaluate the safety and efficacy of self-made polyurethane-covered stent implantation in the emergent scenarios of CAP.

As we know, main vessel perforations can be sealed by CS implantation, whereas distal vessel and collateral vessel perforations can be administered by CS in selected cases. Often, they are promptly treated with fat or coil embolization. But sometimes, deployment of CS across the origin of the perforated side branch and collateral channel can be considered as the last resort option [30]. From our clinical experience, two PU-CS were deployed on the main branch of the perforated vessels and one of them experienced referral to life-saving surgery. Another two CS were implanted directly in the perforated sites and each of them encountered revascularization or total in-stent occlusion. Thus, it seems that the utilization of CS in distal or collateral vessels may have a higher likelihood of adverse events. Given that the number of PU-CS in such situations was limited, conclusions should be drawn with caution. Furthermore, we need to collect more clinical data on the application range of CS and bring benefits to more severe CAP.

## 5. Limitations

The present study possesses some limitations affecting the generalizability of our results. Firstly, it is derived from a single-center retrospective study, sharing all the intrinsic limitations of this kind of design. Secondly, the sample size was small owing to the rare incidence of CAP and emergent nature. Thirdly, not all the patients underwent routine repeat coronary angiograms after long-term follow-up, and therefore we could not accurately assess the rates of ISR/ST.

## 6. Conclusions

Among patients with life-threatening CAP, the utilization of self-made PU-CS demonstrates high rates of successful delivery and sealing of severe CAP during PCI. Although the in-hospital mortality remains high after PU-CS implantation, the long-term follow-up shows favorable clinical outcomes, indicating the feasibility of PU-CS in treating CAP.

## Figures and Tables

**Table 1:** Baseline clinical characteristics.

	Patients (*n* = 24)
Age (years)	68.7 ± 8.4
Male sex (%)	20 (83.3%)
Hypertension (%)	18 (75.0%)
Dyslipidemia (%)	3 (12.5%)
Diabetes mellitus (%)	5 (20.8%)
Current smoking (%)	5 (20.8%)
Current drinking (%)	6 (25.0%)
Previous MI (%)	3 (12.5%)
Previous PCI (%)	17 (70.8%)
Previous CABG (%)	1 (4.2%)
Clinical presentation	
Stable angina (%)	10 (41.7%)
Unstable angina (%)	8 (33.3%)
STEMI (%)	2 (8.3%)
NSTEMI (%)	3 (12.5%)
Silent ischemia (%)	1 (4.2%)
LVEF (%)^a^	58.4 ± 10.0

Data are shown as absolute numbers and percentage (%) or mean ± standard deviation. MI: myocardial infarction; PCI: percutaneous coronary intervention; CABG: coronary artery bypass grafting; STEMI: ST-segment elevation myocardial infarction; NSTEMI: non-ST-segment elevation myocardial infarction; LVEF: left ventricular ejection fraction. ^a^Data are available for 22 (91.7%) of patients.

**Table 2:** Lesion and procedural characteristics.

	Patients (*n* = 24)
*Perforation site*	
Main vessel perforation	
LAD (%)	10 (41.7%)
LCX (%)	5 (20.8%)
RCA (%)	5 (20.8%)
Distal artery wire perforation (%)	2 (8.3%)
Collateral vessel perforation (%)	2 (8.3%)

*Perforation grade (Ellis grade)*	
II (%)	7 (29.2%)
III (%)	17 (70.8%)

*Lesion complexity*	
Types B2 and C (%)	22 (91.7%)
Chronic total occlusion (%)	14 (58.3%)
Calcification lesion (%)	10 (41.7%)
Bifurcation lesion (%)	8 (33.3%)
Torturous lesion (%)	3 (12.5%)

*Device causing perforation*	
Balloon postdilatation (%)	8 (33.3%)
Stent (%)	5 (20.8%)
Balloon predilatation (%)	4 (16.7%)
Rotational atherectomy (%)	4 (16.7%)
Guide wire (%)	3 (12.5%)
IVUS (%)	3 (12.5%)
Guide extension catheter (%)	4 (16.7%)
Successful perforation sealing (%)	19 (79.2%)

*Self-made CS details (n* *=* *31 stents)*	
Average number of CS implanted	1.3 ± 0.5
CS diameter (mm)	2.7 ± 0.4
CS length (mm)	21.1 ± 6.8

Data are shown as absolute numbers and percentage (%) or mean ± standard deviation. LAD: left anterior descending coronary artery; LCX: left circumflex coronary artery; RCA: right coronary artery; CS: covered stent; IVUS: intravascular ultrasound.

**Table 3:** In-hospital clinical outcomes.

	Patients (*n* = 24)
Emergent events during procedure	
Cardiac tamponade (%)	9 (37.5%)
Pericardiocentesis (%)	8 (33.3%)
Emergent surgical repair and CABG (%)	4 (16.7%)
All-cause death (%)	4 (16.7%)
Cardiac death (%)	4 (16.7%)
MI (%)	0
Ischemia-driven TLR (%)	0
ST (%)	0
Hospital stay (days)	8.3 ± 6.2

Data are shown as absolute numbers and percentage (%) or mean ± standard deviation. CABG: coronary artery bypass grafting; MI: myocardial infarction; TLR: target lesion revascularization; ST: stent thrombosis.

**Table 4:** Clinical outcomes at follow-up.

	Patients (*n* = 22)
All-cause death (%)	5 (22.7%)
Cardiac death (%)	5 (22.7%)
MI (%)	0
Ischemia-driven TLR (%)	1 (4.5%)
MACE	6 (27.3%)

Data are shown as absolute numbers and percentage (%). MI: myocardial infarction; TLR: target lesion revascularization; MACE: major adverse cardiac events.

## Data Availability

The data used to support the findings of this study are available from the corresponding author upon request.

## Supplementary Materials

Supplementary Materials Supplementary Table 1: details of all the patients receiving self-made PU-CS implantation in the present study (PU-CS = polyurethane-covered stent). Supplementary Video 1: detailed live example of manufacturing method of self-made PU-CS (PU-CS = polyurethane-covered stent). Supplementary Video 1 can be found online at OneDrive (https://1drv.ms/v/s!Av8xktjr8bcPhSy9WwyepYsyoB2s?e=aceVPv).
