# Supplementary material for: Clinical Outcomes of Self-Made Polyurethane-Covered Stent Implantation for the Treatment of Coronary Artery Perforations
Source: J Interv Cardiol. 2021 May 17;2021:6661763. doi: 10.1155/2021/6661763 (PMC8143889; doi:10.1155/2021/6661763)
Supplement: Supplementary Materials — Supplementary Table 1: details of all the patients receiving self-made PU-CS implantation in the present study (PU-CS = polyurethane-covered stent). Supplementary Video 1: detailed live example of manufacturing method of self-made PU-CS (PU-CS = polyurethane-covered stent). Supplementary Video 1 can be found online at OneDrive (https://1drv.ms/v/s!Av8xktjr8bcPhSy9WwyepYsyoB2s?e=aceVPv). [file 6661763.f1.zip › 6661763.f1/Supplementary Table 1-Revised (1).docx]

| Supplementary Table 1: Details of all the patients receiving self-made PU-CS implantation in the present study. | | | | | | | | |
| --- | --- | --- | --- | --- | --- | --- | --- | --- |
| Patient | Perforation grade | Reason for perforation | Perforation site | CS number | CS size | Cardiac tamponade | Emergent surgery | Cardiac death |
| NO.1 | II | Balloon post-dilation | Middle LAD | 1 | 2.5×16mm | Yes | No | // |
| NO.2 | II | Balloon post-dilation | Middle LCX | 1 | 2.25×18mm | No | No | No |
| NO.3 | II | Balloon post-dilation | Collateral | 1 | 2.5×18mm | Yes | No | No |
| NO.4 | II | Balloon  pre-dilation | Middle LCX | 1 | 2.25×28mm | No | No | No |
| NO.5 | II | RA | Middle LAD | 1 | 2.75×18mm | No | No | No |
| NO.6 | II | RA | Middle LAD | 2 | 3.0×23mm 3.0×29mm | No | No | No |
| NO.7 | II | Guidewire | Diagonal branch | 2 | 2.5×22mm 2.5×23mm | No | No | No |
| NO.8 | III | Balloon post-dilation | Proximal LAD | 1 | 3.0×33mm | Yes | Yes | Yes |
| NO.9 | III | Balloon post-dilation | Middle LAD | 1 | 3×13mm | Yes | No | Yes |
| NO.10 | III | Balloon post-dilation | Middle LAD | 2 | 2.25×23mm 2.5×23mm | Yes | Yes | Yes |
| NO.11 | III | Balloon post-dilation | Middle LCX | 1 | 2.5×13mm | No | No | // |
| NO.12 | III | Balloon post-dilation | Middle RCA | 1 | 4×16mm | No | No | No |
| NO.13 | III | Balloon pre-dilation | Middle LAD | 1 | 2.5×23mm | No | No | Yes |
| NO.14 | III | Balloon pre-dilation | Middle RCA | 1 | 3×38mm | Yes | No | No |
| NO.15 | III | Balloon pre-dilation | Middle RCA | 2 | 2.5×19mm 2.75×19mm | Yes | Yes | No |
| NO.16 | III | Stent implantation | Middle LAD | 1 | 2.5×13mm | No | No | No |
| NO.17 | III | Stent implantation | Proximal LCX | 1 | 2.5×13mm | No | No | No |
| NO.18 | III | Stent implantation | Middle LCX | 2 | 2.5×13mm 2.5×23mm | No | No | No |
| NO.19 | III | Stent implantation | Proximal RCA | 1 | 3.5×23mm | No | No | No |
| NO.20 | III | Stent implantation | Middle RCA | 1 | 3.5×29mm | No | No | No |
| NO.21 | III | RA | Middle LAD | 1 | 2.5×28mm | Yes | No | Yes |
| NO.22 | III | RA | Middle LAD | 2 | 3.0×29mm | No | No | No |
| NO.23 | III | Guidewire | Diagonal branch | 2 | 2.75×13mm | Yes | Yes | No |
| NO.24 | III | Guidewire | Collateral | 1 | 2.5×15mm | No | No | No |
| Abbreviations: PU-CS, polyurethane-covered stent; RA, rotational atherectomy; LAD, left anterior descending coronary artery; LCX, left circumflex coronary artery; RCA, right coronary artery; MI, myocardial infarction; ISR, in-stent restenosis; TLR, target lesion revascularization; ST, stent thrombosis. | | | | | | | | |

| Supplementary Table 1: Details of all the patients receiving self-made PU-CS implantation in the present study. (continued) |
| --- |

| Patient | MI | Ischemia-driven TLR | ST | ISR | Telephone follow-up | Telephone follow-up duration | Angiographic follow-up | | Angiographic follow-up duration |
| --- | --- | --- | --- | --- | --- | --- | --- | --- | --- |
| NO.1 | // | // | // | // | No | // | No | | // |
| NO.2 | No | No | No | No | Yes | 684 days | Yes | | 420 days |
| NO.3 | No | No | // | // | Yes | 246 days | No | | // |
| NO.4 | No | No | No | No | Yes | 744 days | Yes | | 369 days |
| NO.5 | No | No | No | Yes | Yes | 984 days | Yes | | 175 days |
| NO.6 | No | No | No | No | Yes | 564 days | Yes | | 344 days |
| NO.7 | No | No | No | Yes | Yes | 186 days | Yes | | 105 days |
| NO.8 | // | // | // | // | // | // | // | | // |
| NO.9 | // | // | // | // | // | // | // | | // |
| NO.10 | // | // | // | // | // | // | // | | // |
| NO.11 | // | // | // | // | No | // | No | | // |
| NO.12 | No | No | No | No | Yes | 884 days | Yes | | 903 days |
| NO.13 | // | // | // | // | // | // | // | | // |
| NO.14 | No | No | // | // | Yes | 242 days | No | | // |
| NO.15 | No | No | // | // | Yes | 221 days | No | | // |
| NO.16 | No | No | No | No | Yes | 1857 days | | Yes | 651 days |
| NO.17 | No | No | No | No | Yes | 656 days | | Yes | 343 days |
| NO.18 | No | No | // | // | Yes | 857 days | | No | // |
| NO.19 | No | No | No | No | Yes | 319 days | | Yes | 357 days |
| NO.20 | No | No | // | // | Yes | 226 days | | No | // |
| NO.21 | // | // | // | // | Yes | // | | // | // |
| NO.22 | No | No | No | No | Yes | 725 days | | Yes | 384 days |
| NO.23 | No | No | // | // | Yes | 718 days | | No | // |
| NO.24 | No | Yes | // | // | Yes | 263 days | | No | // |
| Abbreviations: PU-CS, polyurethane-covered stent; RA, rotational atherectomy; LAD, left anterior descending coronary artery; LCX, left circumflex coronary artery; RCA, right coronary artery; MI, myocardial infarction; ISR, in-stent restenosis; TLR, target lesion revascularization; ST, stent thrombosis. | | | | | | | | | |
