# Supplementary material for: Clinical Outcomes of Self-Made Polyurethane-Covered Stent Implantation for the Treatment of Coronary Artery Perforations
Source: J Interv Cardiol. 2021 May 17;2021:6661763. doi: 10.1155/2021/6661763 (PMC8143889; doi:10.1155/2021/6661763)
Supplement: Supplementary Materials — Supplementary Table 1: details of all the patients receiving self-made PU-CS implantation in the present study (PU-CS = polyurethane-covered stent). Supplementary Video 1: detailed live example of manufacturing method of self-made PU-CS (PU-CS = polyurethane-covered stent). Supplementary Video 1 can be found online at OneDrive (https://1drv.ms/v/s!Av8xktjr8bcPhSy9WwyepYsyoB2s?e=aceVPv). [file 6661763.f1.zip › 6661763.f1/Supplementary Video 1.docx]

Supplementary Video 1 can be found online at OneDrive：

https://1drv.ms/v/s!Av8xktjr8bcPhSy9WwyepYsyoB2s?e=aceVPv
